# Managing the Marine Aquarium Trade: Revealing the Data Gaps Using Ornamental Polychaetes

**DOI:** 10.1371/journal.pone.0029543

**Published:** 2012-01-03

**Authors:** Joanna M. Murray, Gordon J. Watson, Adriana Giangrande, Margherita Licciano, Matt G. Bentley

**Affiliations:** 1 Institute of Marine Sciences, School of Biological Sciences, University of Portsmouth, Portsmouth, UK; 2 Department of Biological and Environmental Sciences and Technologies, University of Salento, Lecce, Italy; 3 Dove Marine Laboratory, School of Marine Science and Technology, University of Newcastle upon Tyne, Newcastle upon Tyne, UK; University of Sydney, Australia

## Abstract

The marine aquarium industry has great potential to generate jobs in low-income coastal communities creating incentives for the maintenance of a healthy coral reef, if effectively managed. In the absence of current monitoring or legislation to govern the trade, baseline information regarding the species, number and source location of animals traded is missing despite being critical for its successful management and sustainability. An industry assessment to establish the number and provenance of species of ornamental polychaetes (sabellids and serpulids) traded was undertaken across UK wholesalers and retailers. Six geographical regions exporting fan worms were identified. Singapore contributed the highest percentage of imports, but of only one worm “type” whereas Bali, the second largest source, supplied five different worm “types”. Over 50% of UK retailers were supplied by one wholesaler while the remainder were stocked by a mixture of one other wholesaler and/or direct imports from the source country. We estimate that up to 18,500 ornamental polychaetes (16,980 sabellids and 1,018 serpulids) are sold annually in the UK revealing a drastic underestimation of currently accepted trade figures. Incorrect identification (based on exporting region or visual characteristics) of traded animals exacerbates the inaccuracy in market quantification, although identification of preserved sabellids using published keys proved just as inconclusive with high within-species variability and the potential for new or cryptic species. A re-description of the polychaete groups traded using a combination of molecular and morphological techniques is necessary for effective identification and market quantification. This study provides the first assessment of ornamental polychaetes but more importantly highlights the issues surrounding the collection of baseline information necessary to manage the aquarium trade. We recommend that future management should be community based and site-specific with financial and educational support from NGOs, local governments and industry members.

## Introduction

Marine ornamental species have been traded globally since the 1930s but during the last two decades market demand has increased establishing a multi-million dollar industry [1], [2], [3]. Over two million people keep marine aquaria [4] and developing technologies and improved understanding of species' biology is predicted to facilitate further growth [5]. The majority (90%–99%) of ornamentals are obtained from coral reefs with about 45 countries including: Brazil, Maldives, Vietnam, Sri Lanka, Hawai'i, the Caribbean, and the principal suppliers, Indonesia and the Philippines [1], [6] supplying the market. The import market is dominated by the USA contributing 60% of global demand. Western Europe, Japan, and Australia contribute the remainder, although the market is expanding globally [5].

As a low volume but high value market, the ornamental trade has the potential to provide invaluable economic stability for rural, low-income coastal communities that supply the trade [7]. However, industry monitoring and reporting is not sufficiently developed although fundamentally important in its management [8]. Trade figures are frequently underreported due to the exclusion or misclassification of shipment records [9] and where records are available, they are commonly classified in weight or value as opposed to number of individuals [2]. The Convention of International Trade in Endangered Species (CITES) protects a number of ornamental hard corals [10] and clams (*Tridacna spp.*) but for the majority of aquarium species it is not known whether they are at risk of exploitation and, therefore, CITES monitoring is an inappropriate tool for management of the industry as a whole.

Currently, the only available source of quantitative data to monitor the marine aquarium trade are business sales records and invoices, as were used in this study, with the exception of Florida which requires all collected fisheries products to be reported. In April 2000, the UNEP-World Conservation Monitoring Centre (UNEP-WCMC) and the Marine Aquarium Council (MAC) established the Global Marine Aquarium Database (GMAD). The database is freely available online and was the first attempt at standardising data from 45 representative wholesale exporters and importers. The database is however not without its limitations. Most importantly it relies on volunteered information with only one fifth of wholesalers submitting data [11] and although they were asked to utilize scientific nomenclature for recording livestock, common English names were often used due to a poor level of standard taxonomy. Finally, funding for GMAD ended in 2004 leaving the database outdated and without a replacement system.

During the last decade, there has been a shift in hobbyist's preference from fish-only tanks to mini-reefs based on a live-rock framework, structural corals and a range of invertebrate species [9], [12]. Their increasing popularity has driven a rise in the diversity and quantity of invertebrates traded. Best estimates predict that between 9 and 10 million individuals, of more than 500 different species (excluding corals), are sold globally although a poor standard of taxonomy makes arriving at an exact figure problematic [4]. Actual numbers traded are however thought to be much higher [4] and a recent study by Rhyne et al [12] found a 10-fold increase in landings of ornamental invertebrates from the Florida Marine Life Fishery between 1994 and 2007 equating to over 500,000 individuals per year.

A variety of segmented worms can be found in the reef aquarium but only sabellids (fan worms) and serpulids (coco worms) are specifically harvested. Accurate taxonomic identification of sabellids requires the removal of the worm from its tube (which can be fatal) and, therefore, confusion at the species level abounds. Morphological characters used to distinguish between species have also shown a great deal of intra-specific variability which makes species boundaries problematic to ascertain [13], [14].

According to GMAD, the UK is the second largest importer of marine fan worms with 11,178 individuals imported between 1991 and 2001 and 1,652 coco worms between 1996 and 2001. Exporting regions to the UK include; Indonesia, the Philippines, Singapore, the Indian Ocean, Sri Lanka, USA, Brazil, Cuba and Martinique, although like much of the data available on invertebrates, only 32% of UK records are associated with a known export country. Furthermore, 92% of the fan worms entering the UK were reportedly *Sabella pavonina* from an unknown exporting region. *S. pavonina* is distributed in the eastern Atlantic and is particularly common in northwest Europe [15] but is almost certainly not suitable for tropical reef aquaria.

The production of a management strategy would be hindered by inaccuracies in numbers collected, source location and the taxonomy of traded species. As part of a larger study on the aquaculture of ornamental fan worms, marine aquarium wholesalers and retailers across the UK were surveyed to establish the quantity and origin of sabellids and serpulids traded. To assess the taxonomic validity of the trade names and the diversity of polychaete species available, samples of sabellids imported by the Tropical Marine Centre Ltd (TMC) were collected for identification using scientific keys. Industry responses and data collected were used to measure the value of existing baseline monitoring of the UK's polychaete trade and, ultimately to uncover what information is missing but essential for the future development of an effective management plan.

## Materials and Methods

The Tropical Marine Centre ® (TMC) provided a sales-based dataset of polychaetes imported into the UK between January 2007 and December 2009. Information included: export region, trade name and the numbers imported into the UK for re-distribution. Sabellids and serpulids were selected and returned to the Institute of Marine Sciences, Portsmouth. The TMC classified individuals as “different species” in relation to their appearance or origin for trade purposes and were not based on scientific taxonomy, therefore, the “different species” named by TMC will be referred to as “types” in this paper. Specimens of each type were photographed in aquaria (using a Nikon D50 digital SLR camera) within their tube and branchial crown extended. A detailed analysis of traded sabellids collected from three of the most important regions; Singapore, the Philippines and Kenya was completed to assess the diversity of species within these regions. All individuals were fixed in 4% formalin for taxonomic identification using Knight-Jones and Mackie's [13] revision of *Sabellastarte*. No specific permits were required for the described laboratory studies.

### Retailers: telephone questionnaire

A simple questionnaire was devised for telephone interviews targeting retailers across the UK. The Practical Fishkeeping magazine's 2009 retailer directory was used to provide the most recent information for stores trading in marine stock. Ten stores were selected at random from each region; Scotland, Northern England, the Midlands and Wales together with twenty stores from the south of England as a representation of the retailer trade. It was first established whether or not the store stocked marine ornamentals before presenting a standardised introduction to a member of the management team informing them of the overarching aims of the study and the brevity of the questions to be asked. If the respondents agreed, four short questions were asked: 1. Does your store stock marine ornamental polychaetes?; 2. Does your store stock both fan worms and coco worms?; 3. Based on back-dated invoice records how many of each do you sell per month?; and 4. Where do you purchase your ornamental polychaetes from? The survey format developed for assessing the UK was then transferred to retail stores throughout Germany as an indication of the European market. Telephone numbers of 62 stores in Germany were collected from an internet-based search and the UK introduction and questions translated. Validation was possible as retailer sales information was collected only from management team members who had full access to sales and business records and who could provide a true verbal summary of this data.

## Results

### Marine ornamental polychaetes: the Tropical Marine Centre

TMC imports more than 1200 species of livestock, from 39 suppliers in 26 countries around the world. Approximately 20 shipments arrive at their three UK facilities each week. Six regions were identified as suppliers of polychaetes, but Bali, Singapore and the Philippines jointly accounted for 89% of the 41,664 individuals imported for re-distribution across the UK, Ireland, the Channel Islands and mainland Europe between the start of 2007 and the end of 2009 (Table 1). It is estimated that between 80–90% of livestock imported to TMC is distributed throughout the UK.

**Table 1:** Export location of ornamental polychaetes.

Export Region	Type name	Number Imported	% of Total TMC Imports
**Bali**	Midnight	381	0.9%
	Pink and white	4,760	11%
	Spiral	1,561	4%
	Yellow	1,886	4.5%
	Hard Tube (Serpulid)	2,357	6%
			*Region Total 26%*
**Indian Ocean**	Orange and White	12,851	31%
**Singapore**	Common	13,343	32%
**Dominican Republic**	Cluster duster	1,107	3%
**Philippines**	Caribbean	3,190	7.5%
**Hawai'i**	Giant Hawaiian	228	0.5%

Number and percentage contribution of the different “types” of worms identified by the Tropical Marine Centre from the six exporting regions which supply the TMC's ornamental polychaetes between 2007 and 2009. Data includes total import sales for TMC.

**Figure 1:**
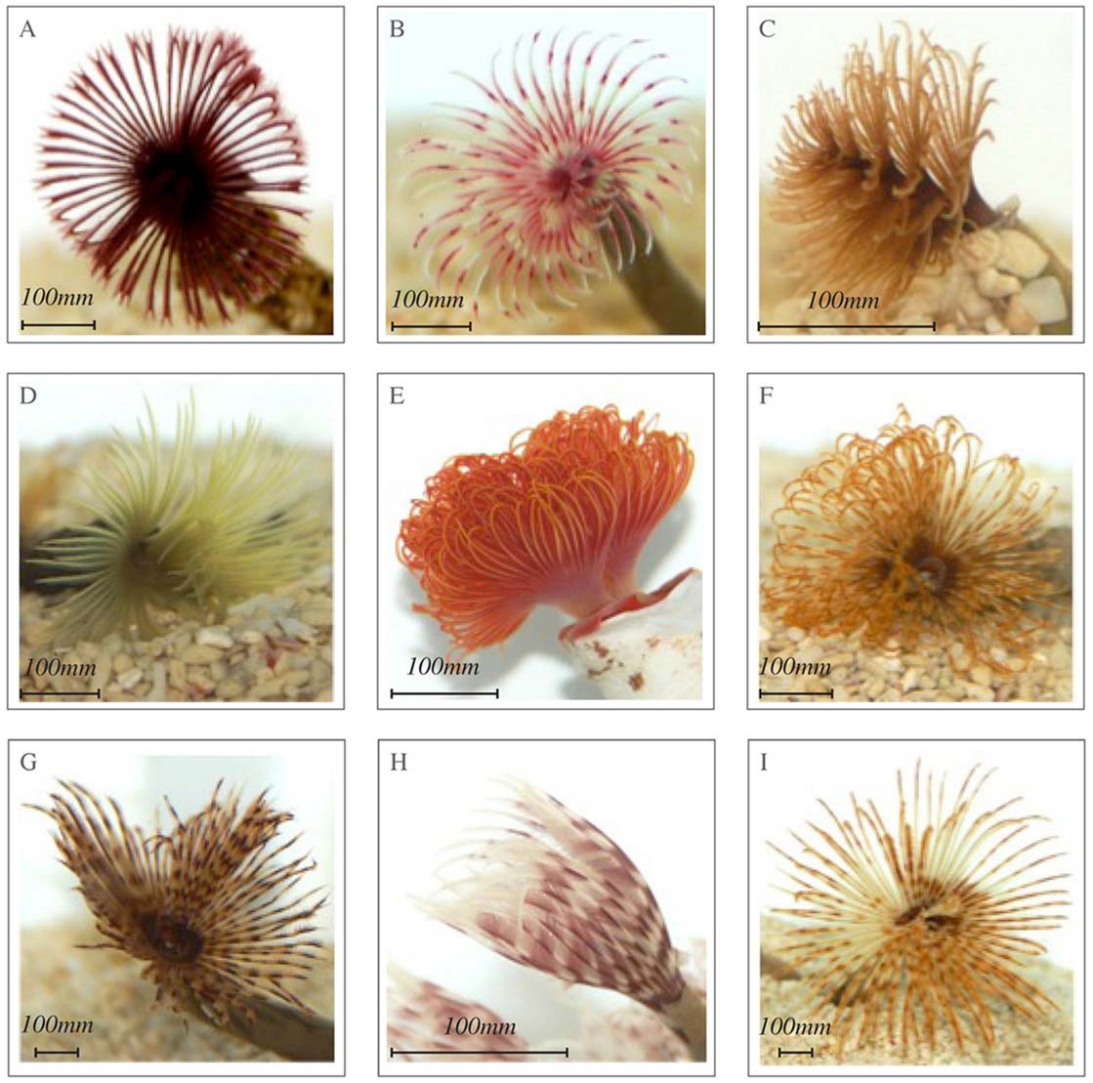
“Types” of tropical polychaetes imported by TMC. “Types” of tropical polychaetes imported into the UK by TMC. a) “Midnight”, b) “Pink and white”, c) “Spiral”, d) “Yellow”, e) “Hard tube” from Bali; f) “Orange and white” from the Indian Ocean, g) “common” from Singapore, h) “cluster duster” from Dominican Republic, and i) “Caribbean” from the Philippines (photographs by J. Murray).

Six “types” were imported from Bali and included; “hard tube worm”, “midnight”, “pink and white”, “spiral”, “yellow” and “indo”. Only the “cluster duster” was imported from the Dominican Republic and only one “type” was imported from Singapore and the Philippines, named the “common feather duster” and “Caribbean” respectively (Figure 1). The “common feather duster” from Singapore was the most frequently traded worm while Bali had the highest diversity in terms of the number of different types it exports. Hawai'i contributed the lowest number of individuals with only 183 “giant Hawaiians” imported in a three year period. Only Bali supplied “hard tube” or coco worms with 2,357 individuals traded during the study.

Identification of individuals of *Sabellastarte* imported from Singapore, the Philippines and Kenya was not possible in the current study as specimens from all regions had a mixture of characters. Therefore, a series of 12 working groups (A–L) were derived based on the presence or absence of similar morphological characters [13] and the species of *Sabellastarte* to which the group was most similar was assigned (Table 2). Specimens from the Philippines exhibited the greatest diversity being allocated to five different groups and showed the greatest diversity of species of *Sabellastarte* to which they were listed as similar; *S. spectabilis*, *S. fallax*, *S. samoensis*, and *S. santijosephi*. Specimens from Kenya and Singapore were allocated to four of the twelve devised groups, however, all Kenyan specimens were found to be similar to either *S. samoensis* or *S. santijosephi* and specimens from Singapore were most similar to *S. samoensis* or *S. spectabilis*.

**Table 2:** The collection location and morphological features of *Sabellastarte* species traded in the UK.

TMC “type” name	Region collected	Group	Key characters	Conclusion	Known distribution
Common	Singapore (2)	**A**	Short *thx* and short *dl*, no undulations on *rds*	Cf. *Sabellastarte samoensis*	Possibly endemic to Samoan region
Common	Singapore (2)	**B**	Short *thx*, short long *dl*, *bc* not separated-short *wb* andpockets present.	Cf. *Sabellastarte spectabilis*	Indo Pacific Oceans
Common	Singapore (3)	**C**	*Bc* about half body length, *c* with pockets and notches, long emergent part on *thx ch*, narrower shaft than knee on *ab ch*, long *dl*, *vcl* overlap (maybe squashed).Square shaped rds.		
Common	Singapore (10)	**D**	*Sabellastarte spectabilis* characters with differences in the *c* between specimens. Possibly intra-specific variation.		
Caribbean	Philippines (5)	**E**	Short thx (as long as wide), short *bc* to body length- *bc* quite spiralled, long *dl*, *bc* not separated with undeveloped notches, *rds* with longitudinal ridges.		
Caribbean	Philippines (2)	**F**	Like group E but with short *dl*.		
Caribbean	Philippines (2)	**G**	Short thx, short *bc*- circled, *dl* about 1/3 of *bc* length, short emergent part on the ch (maybe contracted on fixing), doubled ridge along the length of *rds*, short *rds* tip (shorter than that of *spectablilis*), c without notches but with deep *cp*. *C* always meets at the midline and joins here (similar to the *c* from **E**).	Cf. *Sabellastarte fallax*	Unknown
Caribbean	Philippines (3)	**H**	Short thx, long emergent part on *thx ch*, shaft narrower than knee on the *ab ch*, long *bc*- at least half body length *rds* with squared edges. *C* is separated.	Cf. *Sabellastarte sanctjosephi*	Possibly confined to the Red Sea
Caribbean and Indian Ocean	Philippines (3); Kenya (1)	**I**	Long *thx*, *bc* about 1/3 of body length with long *dl*, knee same width as the shaft on the *ab ch*, *c* separated and with lappets, shallow pockets, very developed *vcl*, *rds* square in profile with undulations. Only difference between group **I** and **J** are the undulations on the *rds*.	Cf. *Sabellastarte samoensis*	Possibly endemic to Samoan region
Indian Ocean	Kenya (2)	**J**	Long *thx* (longer than wide), long *dl*, short *bc* to body length- circled, *cp* and notches present, bc lobes narrowly separated.		
Indian Ocean	Kenya (1)	**K**	Long *thx*, short *bc* with short *dl*, *c* is embayed- does not have incisions. Knee with a narrower shaft on *ab ch*.		
Indian Ocean	Kenya (1)	**L**	Short thx and *dlb* of *bc* widely separated.	Cf. *Sabellastarte sanctjosephi*	Possibly confined to the Red Sea

Taxonomic groupings (A–L) based on specimens from the Singapore, Philippines and Kenya supplied by TMC. Information listed includes the number of specimens in each group, their locality, defining morphological characters; thorax (*Thx*), dorsal lips (*dl*), radioles (*rds*), branchial crown (*bc*), crown web (*wb*), collar (*c*), thoracic chaetae (*thx ch*), abdominal chaetae (*ab ch*), ventral collar lappets (*vcl*) collar pockets (*cp*) and dorsal lobes (*dlb*), and a conclusion to which species from the genus the group is most closely suited with their known distribution (Knight-Jones and Mackie, 2003). Specimens from the Indo Pacific Ocean includes records from; Zanzibar, Mauritius, Sri Lanka, Burma, Indonesia, Philippines, Japan, Taiwan, Hawaii & Western and Northern Australia.

All 12 of the working groups were likened to one of four species of the eight known in the *Sabellastarte* genus; *S. samoensis*, *S. spectabilis*, *S. fallax* or *S. santijoesphi*. Five of these groups (B, C, D, E, and F) were most similar to *S. spectabilis*. The majority of morphological features examined matched the species description for *S. spectabilis* with only one character notably different, for example, the length of the dorsal lips, the shape and dimensions of the radioles or the arrangement of the collar. It is not clear if these specimens represent different species or simply between-specimen variations.

### Surveying the retailer

**Figure 2:**
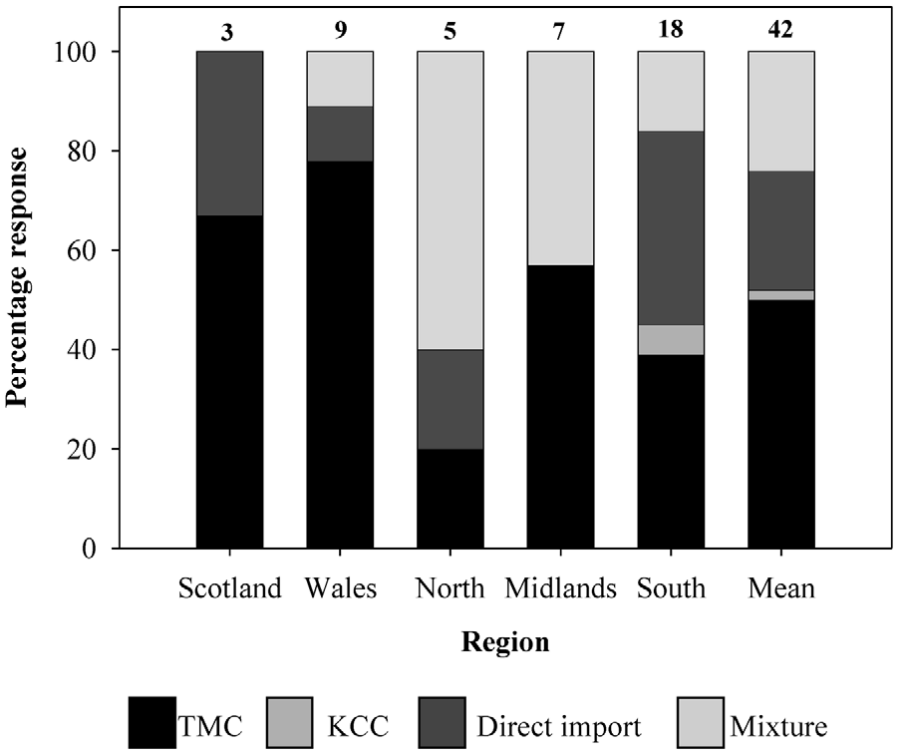
The source of marine ornamental stock in UK retail stores. Percentage of retailers across the UK; Scotland, Wales, the north, midlands and south of England and the UK mean, purchasing marine fan worms from TMC, KCC (a wholesaler based in Hull), importing directly themselves or a mixture of any of these (corresponding shading shown in legend). The number of retail stores that stock fan worms and answered this question is shown above the corresponding data bar.

One hundred percent of stores approached in Wales (n = 10) and the south of England (n = 20), 90% of stores in the north (n = 10) and midlands (n = 10) and 60% of the stores approached in Scotland (n = 5) agreed to take part and answered all telephone survey questions. On all occasions, an un-willingness to participate was attributed to being too busy despite emphasising the briefness of the survey. An average of 82% of surveyed stores stocked marine polychaetes, although the north of England represented the lowest percentage with only 56% of marine stores selling fan worms (Figure 2). Although 100% of the stores surveyed in Scotland stocked marine worms, it should be noted that only three out of five retailers participated (Figure 2). Of the 62 stores surveyed in Germany, only 44 sold marine stock and only 50% of those reported stocking ornamental polychaetes. The range and mean number of fan worms sold by retailers in all regions per month is presented in Table 3. There is considerable variation between retailers and while some stores report only one sale per month, others report selling up to 40. One store in Scotland stated that they sell up to 100 individuals per month and it is this variability which hides between-region differences. In all cases, retailers sold fewer coco worms (10 per month in the UK, 3 per month in Germany) than fan worms (23 per month in the UK, 10 per month in Germany).

**Table 3:** The number of ornamental polychaetes sold in the UK and Germany each month.

	% of marine stores stocking polychaetes	Coco worms			Fan worms		
		Stocked stores	Range of worm sales per month	Mean number sold per month	Stocked stores	Range of worm sales per month	Mean number sold per month
Scotland (3)	100	1	30	30	2	40–100	70
Wales (10)	90	2	1–12	7	9	2–48	11
North (9)	56	1	3	3	4	3–10	7
Midlands (9)	79	2	2–4	3	7	4–50	14
South (20)	90	7	1–12	5	18	3–30	13
**UK Mean** (42)				**10**			**23**
Germany	50	16	1–10	3	19	1–70	10

The estimated range and mean number of coco worms and fan worms sold on a monthly basis in stores participating in the retail telephone survey across the UK; Scotland, Wales and the north, midlands and south of England and Germany. The percentage of retailers selling marine stock is present in brackets next to the corresponding region.

Four sources of stock were identified and included; TMC (London and Bristol), KKC (a wholesaler based in Hull), direct import or a combination of two or more of the previous. TMC was identified as the main supplier to stores in Scotland (67%), Wales (78%) and the midlands (57%) (Figure 2). TMC and direct import equally constituted the largest supply sources to southern stores (39%), 16% of retailers recorded a mixture of categories and only 6% of surveyed stores reported to import exclusively from KKC.

### Calculating the UK trade in polychaetes

Fifty two percent of stores surveyed in the UK purchase stock exclusively from TMC, and a further 24% purchase from a combination of sources including TMC, KKC and direct import. TMC's contribution to the UK's annual sales of ornamental polychaetes (including both coco worms and fan worms) was estimated at 12,499 (90% of the 41,664 polychaetes imported by TMC and distributed within the UK over three years). This, however, does not include the role of other wholesalers (KKC) and direct imports which contribute additional sales. Considering that TMC imports may supply a maximum of 76% of retailers in the UK (52% of stores exclusively purchasing from TMC plus the potential for an addition 24% which may import from TMC in combination with another source) a conservative estimate suggests a potential increase of 24% (retail stores not using TMC at all) on the current annual total. Alternatively, if the “mixture” of sources did not include TMC but relied on direct import and other wholesalers alone, there would be a 48% increase on the total number of polychaetes (12,499) imported by TMC. Based on these calculations, the actual number of ornamental polychaetes traded annually in the UK is estimated at between 15,498 and 18,497 worms per year (between 877–1,046 coco worms and 14,621 and 17,451 fan worms). For a comparison with these values, retailer data on the number of polychaetes sold per month (Table 3) was used to estimate the number of polychaetes sold annually. The mean number of surveyed stores selling fan worms (82%) (Figure 1) was used to calculate that out of the 323 marine stores listed in the Practical Fishkeeping magazine's 2009 retailer directory, approximately 73,101 fan worms and 31, 800 coco worms could potentially be sold annually across the UK (based on an average of 23 and 10 worms respectively being sold at each store every month) (Table 3).

## Discussion

Effective monitoring and regulation of the marine aquarium industry is constrained by a lack of good quality, quantitative and un-biased information [11] and confounded by taxonomic confusion. The source location of imported polychaetes would provide an important clue in their accurate identification; however the collection points provided by TMC (Singapore, the Philippines and Kenya) encompassed vast geographical ranges representing export locations instead of the collection site. Specifically, Singapore is the trading hub of Asia re-exporting aquarium organisms collected throughout the region [9] and therefore individuals from “Singapore” may have originated from numerous localities. This missing point-source location data within the trade at present compromises the validity of polychaete taxonomy and highlights its importance in aiding accurate identification.

It was hypothesised that erroneous taxonomy of ornamental fan worms was fundamentally an industry-level issue due to the practicalities of tube removal for identification. However, this study revealed that the industry is constrained by unclear species boundaries and distributions within the scientific literature which forms the trade's reference material. The scientific taxonomy of sabellids is still far from being understood with a number of groups requiring revision and with species yet to be described. Recently, Capa et al. [14] found diagnostic characters used in the existing *Sabellastarte* key [13] varied greatly within species, and also overlapped between species. Anaesthetization and fixation protocols have been shown to induce pseudo-differences in the gross morphology of some sabellids [16] and Knight-Jones & Mackie [13] expressed particular concern over the distortion of “soft” characters, such as the collar, if the specimen was fixed within its tube. Moreover, their extensive capacity to regenerate [17] is also of concern for achieving accurate identification. The length of the branchial crown in relation to body length is often used in the early stages of the morphological key and relies on the assumption that it is full size at the time of examination when in fact sabellids are known to readily autotomize their crowns following predation or a mechanical stress [18; pers. obs. J. Murray]. Capa et al., [14] have begun to address this issue by combining molecular (COI and 16S) and morphological data in an integrative approach to establish species in the *Sabellastarte* genus.

The current attempt to identify members of *Sabellastarte* supplying the trade also suggests that there may be a number of un-described or cryptic species with a diverse mixture of characters from a number of known species present within an individual (pers. comm. A. Mackie, 2009) and the validity of the 12 working groups used here cannot be supported without the use of molecular tools. Continuation of the work by Capa et al. [14], is critical to establish the species and species-boundaries in *Sabellastarte* before transferring this information to the aquarium trade. The ornamental industry itself, however, offers the potential to facilitate such scientific studies by providing sabellids from various localities across the world and a unique opportunity for a global analysis of sabellid taxonomy. While sabellid taxonomy within the trade will always be limited due to the impracticalities of removing specimens from their tube for identification, the establishment of comprehensive species distribution and boundaries records would allow the trade to better predict which species they have collected, assuming the scientific taxonomy is fully understood and range expansions or displacement are well documented.

This study reveals a drastic underestimation of currently accepted global trade quantities. GMAD's estimate of total numbers of fan worms and coco worms imported annually into the UK was 5,890 worms while in the current study, we found that 12,499 fan worms where imported annually by a single wholesaler alone. When other sources of import were considered (KKC and direct import) the actual number of ornamental polychaetes traded in the UK may be as high as 18,500 individuals per year while based on retailer this number could even be as high as 73,101 per year. It is more than likely that such discrepancies are evident worldwide and with global trade in fan worms between 1988 and 2002 estimated at 9,160 individuals per year [4], less than the TMC's annual UK imports, the degree of underestimation is likely to be substantial. Using the error margin associated with UK records, we can estimate that more than 172,088 fan worms are actually traded worldwide [4]. While the figures obtained in this study clearly indicate that the diversity and volume of traded polychaetes is substantially greater than previously documented, it also highlights the problematic nature of estimating the size of the industry using a top-down approach. These estimations rely on the accuracy of the conveyed wholesaler and retailer sales figures and their willingness to participate; it also does not include the internet trade or take into account any mortality along the line of custody.

In the light of a recent study by Rhyne et al [12] that reported a 10-fold increase over the last 15 years in Florida's ornamental invertebrate catches, and with the increasing number of anthropogenic threats to coral reefs ecosystems [19]; urgent monitoring of all species collected for the aquarium trade is essential to ensure the durability of the future industry. The top-down monitoring approach presented in this paper which surveyed national wholesalers and retailers highlighted a substantial underestimation of the UK's trade in ornamental polychaetes but we believe this monitoring strategy would not be successful trade-wide without substantial investment of resources (which is unlikely). Monitoring of ornamental fisheries should take an inclusive approach from the point of collection using site-specific community-based management (CBM) as a framework. The CBM approach has been shown to be effective for a number of sites [7], [20] incorporating management, education of local collectors and promotion of sustainable methods of collection. Using this method, the quantification of organisms imported or exported in the global trade would be of less importance as the focus would be site-specific with local-scale limits and maximum collection capacities, obviating the difficulties with establishing the source location of traded organisms. CBM was to be integral to the Ecosystem and Fishery Management core standard of the Marine Aquarium Council (MAC) trade-wide certification programme implemented from 2001. However this programme does not seem to have taken hold, at least in the UK. For CBM to be effective it must have scientific input, stakeholder participation and incentives. It is not clear why MAC certification has not been more successful despite significant stakeholder involvement but a lack of consumer awareness and a failure of the incentive system (‘a green price premium’) for industry members are likely factors.

Obtaining accurate taxonomy is not just problematic for polychaetes, but one which extends to most traded invertebrates [4] and improving scientific understanding of their taxonomy must be a priority goal. The advent of DNA barcoding and other molecular techniques in combination with morphologically-based taxonomy may offer a relatively inexpensive method for scientists to address this issue and has already been used for marine fishes [21]. Still, even if the scientific literature can be improved and this information relayed to industry members, reliable identification of target species would remain problematic as the accuracy of non-specialists identifying them has been shown to be poor [10] and as the majority of consumers are disinterested in knowing the scientific name of their purchase, incentives to use correct nomenclature is low. In the short term it may prove more beneficial to focus at the ecosystem function level and monitor, for example, the number of filter-feeding sabellids or grazing top shells which are collected from a site.

Aquaculture of ornamental species is a rapidly growing sector and often seen as a priority solution in the conservation of reef habitats, relieving collection pressures on wild stocks. However, only 1–10% of marine ornamental fish are commercially bred and the number of invertebrates successfully reared in captivity is low and limited to a few species. Expanding the range of invertebrates cultured is problematic and constrained by bottlenecks at key stages in life histories under current technologies [4]. Furthermore, due to the problematic nature of rearing marine ornamental larvae, many captive bred projects are found in developed countries that have both the facilities and infrastructure to support such ventures [22]. As the second most collected ornamental species within the state of Hawai'i [23] there has been a drive in research efforts to understand the reproduction and life history of *Sabellastarte spectabilis* to facilitate its future culture [24], [25], [26], [27]. Despite this, propagation is reliant on the initial collection of wild-caught stock and taking c. 200 days to reach marketable size (10 mm tube diameter) [23], the scale-up of this research to commercial production is still some way off. To monitor the marine aquarium trade, a collection-point focused strategy, supported by local governments, NGOs and industry stakeholders from all levels, providing communities with scientific and management expertise, direct financial support and education on the benefits of coral reef stewardship, should be the immediate goal to ensure the sustainable future of the marine aquarium trade in the context of wider coral reef management issue.
